# High-throughput investigation of macromolecular interactions for drug development using spectral shift technology

**DOI:** 10.1007/s12551-025-01359-x

**Published:** 2025-09-03

**Authors:** Charlotte E. Hunter, Ehmke Pohl, Stefanie Freitag-Pohl

**Affiliations:** 1 Department of Chemistry, Durham University, South Road, Durham, DH1 3LE UK; 2 Biophysical Sciences Institute, Durham University, South Road, Durham, DH1 3LE UK

**Keywords:** Macromolecular interactions, Biophysical assays, Drug design, Spectral shift, TRIC, Dianthus

## Abstract

This review focuses on spectral shift analysis as a tool to study macromolecular interactions and describes its current place among the available biophysical methods. NanoTemper’s Dianthus platform facilitates a plate-based, microfluidics-free, mass-independent, and immobilisation-free high-throughput screening platform for protein–ligand, protein–protein, and protein–nucleic acid interactions, as well as ternary complexes, for example in proteolysis targeting chimera (PROTAC) design. In addition to spectral shift, the Dianthus offers an orthogonal method, temperature-related intensity change (TRIC). Both methods are presented alongside fluorescent labelling techniques. Specific examples with practical tips for spectral shift methods for diverse binding partners are provided. Finally, current and future applications of spectral shift methods in the drug discovery process are discussed in the context of high-throughput screening, fragment-based drug discovery, and hit-to-lead optimisation.

## Introduction

### The importance of biomolecular interactions

Macromolecular interactions are a cornerstone of all biological processes and thus a key consideration in drug development. These interactions, ranging from protein–ligand binding to complex multimeric assemblies, need to be characterised by robust assays, as the drug discovery process is often laborious and expensive (average of 6–10 years, $0.2–4.5 billion per drug development case (Schlander et al. 2021)). Many biological assays are dependent on the enzymatic function of a protein and thus on the kinetic mechanism of its binding to another molecule. Biophysical binding assays observe physicochemical properties, such as changes in fluorescence that depend directly on macromolecular interactions. This is often advantageous, especially in identifying false hits and weak binders in fragment screening. Traditional biophysical binding techniques like surface plasmon resonance (SPR), nuclear magnetic resonance (NMR), and isothermal titration calorimetry (ITC) have been instrumental but often face limitations, requiring surface immobilisation, high sample and time consumption, or complex assay development. NMR has long been the approach to library screening (Shuker et al. 1996; Hajduk and Burns 2002) due to its reliability and information content (Keiffer et al. 2020); however, other techniques have been developed to overcome some of its biggest limitations: high sample consumption and limited throughput. SPR and thermal shift techniques have covered this increase in throughput to some degree, requiring less target protein, but these methods are intrinsically noisier (Holdgate et al. 2013).

This array of challenges becomes particularly pronounced during early-stage drug discovery, where large libraries must be screened ideally under native-like conditions. Often, these libraries consist of small chemical fragments that are difficult to screen with methods that depend on considerable mass change and often cannot detect weak binding. The need to accurately estimate the affinity of binders is crucial in deciding which compounds are most likely to succeed. NanoTemper Technologies has recently introduced the Dianthus platform (NanoTemper Technologies 2019), an immobilisation-free, mass-independent, automation-compatible, microfluidics-free, plate-based system. Built on the principles of spectral shift (SpS) and temperature-related intensity change (TRIC), the Dianthus offers sensitive, versatile, and high-throughput affinity-based characterisation of molecular interactions. This addresses the growing challenges in modern drug discovery, particularly for difficult targets, such as intrinsically disordered proteins (IDPs) (Qin et al.), targeted protein degradation (TPD), including proteolysis targeting chimeras (PROTACs) (Li et al. 2022), and multimeric assemblies (Xie et al. 2023). This review delves into the biophysical principles underpinning the Dianthus screening platform, compares it to traditional technologies, and evaluates its utility across various stages of drug discovery. In addition, we demonstrate the capability of spectral shift to quantify high-affinity interactions as well as protein–protein interactions.

### Overview of biophysical techniques for macromolecular interaction analysis

**Fig. 1:**
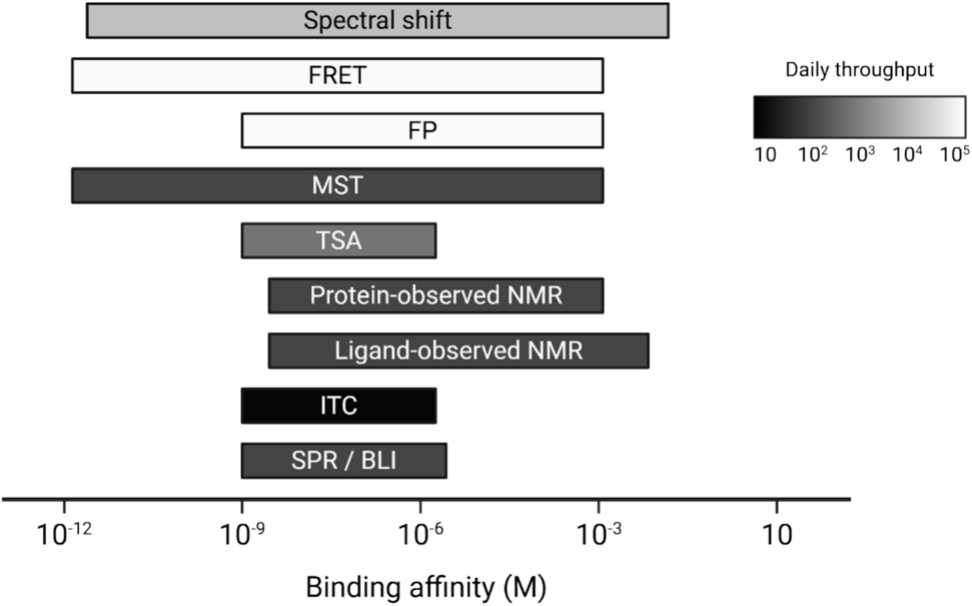
Binding affinity limits and throughput of various biophysical techniques, references are shown in Table 1 (created in BioRender; https://BioRender.com/eag6ljm)

The field of biophysical analysis of molecular interactions is rich with techniques that have evolved to meet diverse demands. While effective, these techniques differ in their underlying physical principles and offer distinct advantages, but each suffers from critical limitations that influence their applicability in different contexts. They often involve immobilisation of one interaction partner, require large sample volumes, or are limited in throughput. Comprehensive reviews on these techniques and their comparisons are readily available (Holdgate et al. 2013; Du et al. 2016; Renaud et al. 2016; Biswas 2018; Kumar et al. 2024; Zhao et al. 2024); see Table 1 and Fig. 1 for a brief overview of some of the most widely used biophysical technologies and their respective strengths and limitations.

**Table 1:** Overview of conventional affinity-based biophysical methods for studying macromolecular interactions

Method	Principle	Affinity range	Daily throughput	Strengths	Challenges	References
SPR (surface plasmon resonance)	Measures changes in refractive index of a sensor surface with immobilised target as binding occurs	1 nM – 500 µM	10^2^	Label-free	Immobilisation required: may affect biological function	(Acharya et al., 2024; Bakhtiar, 2013; Cooper, 2002; de Mol & Fischer, 2008; Giannetti, 2011; Kortt et al., 1997; Myszka et al., 1998; Olaru et al., 2015; Schuck & Zhao, 2010; Wijaya et al., 2011)
				Real-time measurement of kinetic and affinity data		
					Fluidics-based: frequent maintenance	
					Affected by non-specific binding and solvent effects	
					Complex assay development	
BLI (biolayer interferometry)	Measures interference pattern of white light reflected from sensor surface with immobilised target as binding occurs	1 nM – 500 µM	10^3^	Label-free	Immobilisation required: may affect biological function	(Abdiche et al., 2008; Concepcion et al., 2009; Jug et al., 2024; Murali et al., 2022; Weeramange et al., 2020)
				Real-time measurement of kinetic and affinity data		
					Affected by non-specific binding Low sensitivity for small molecules	
					Poor reproducibility	
ITC (isothermal titration calorimetry)	Measures heat changes during binding events in solution	1 nM – 100 µM	10	Label-free	Requirement for compound purity due to high sensitivity	(Ladbury & Chowdhry, 1996; Leavitt & Freire, 2001; Lewis & Murphy, 2005)
				Immobilisation-free		
				Direct measurement of binding affinity and thermodynamics	Large sample consumption	
					Time-consuming	
				Highly sensitive		
Protein-observed NMR	Changes in spectra	100 nM – 1 mM	10^2^	Immobilisation-free	Very large amounts of isotopically labelled protein required	(Gossert & Jahnke, 2016; Lamoree & Hubbard, 2017; Lepre, 2011; Lepre et al., 2004)
				Can determine binding site		
					Limit on protein molecular mass to ~ 40 kDa	
Ligand- observed NMR	Changes in spectra	100 nM – 10 mM	10^2^	Immobilisation-free	Requires large amounts of protein	(Lamoree & Hubbard, 2018; Lepre, 2011)
				Monitor ligand and protein state / degradation	Cannot determine Kd	
				High upper limit of binding affinity		
Analytical ultracentrifugation AUC	Observes sedimentation profile with optical detection systems	1 pM – 100 mM	10	Immobilisation-free	Labelling for high affinity	(Harding & Rowe, 2010)
					Time-consuming	
					Limited to protein-protein, protein-DNA interactions	
TSA (thermal shift assay) / DSF (differential scanning fluorimetry)	Monitors protein melting temperature changes upon binding	1 nM – 100 µM	10^3^	Immobilisation-free	Requirement of dye or high intrinsic fluorescence	(Alexander et al., 2014; Brandts & Lin, 1990; Bruce et al., 2019; Chattopadhyay & Varadarajan, 2019; Gao et al., 2020; Kranz & Schalk-Hihi, 2011; Lo et al., 2004; Pantoliano et al., 2001; Semisotnov et al., 1991)
				Label-free		
				Well-established method, uses basic lab equipment	Convoluted by protein-dye interaction / protein unfolding	
					Likelihood of false positives and negatives is high	
					Indirect method, no kinetic data	
MST (microscale thermophoresis)	Detects changes in molecular motion in a temperature gradient upon binding	1 pM – 1 mM	10^2^	Immobilisation-free	Fluorescent labelling typically required	(Baaske et al., 2018; Duhr & Braun, 2006; Jerabek-Willemsen et al., 2014; Seidel et al., 2013; Wienken et al., 2010)
				High sensitivity		
				Can use complex systems without the need for purification		
				Low sample consumption		
Fluorescence polarisation (FP)	Measures rotational diffusion changes upon ligand binding	1 nM – 1 mM	10^5^	Immobilisation-free	Requires ligand fluorophore labelling	(Hall et al., 2016; Huang, 2003; Kumar et al., 2024)
				High throughput		
					Limited to small ligand-large target size changes	
Förster resonance energy transfer (FRET)	Measures energy transfer between two fluorophores in close proximity	1 pM – 1 mM	10^5^	Immobilisation-free	Requires precise fluorophore labelling for both binding partners	(Degorce, 2009; Kumar et al., 2024; Stryer & Haugland, 1967)
				High sensitivity and resolution		
					Limited to small ligand-large target size changes	
SpS (Spectral shift)	Measures the emission spectrum shifts of an environmentally sensitive dye as a result of ligand binding	250 pm – 20 mM	10^3^ - 10^5^	Immobilisation-free	Requires fluorophore labelling	(Baaske & Langer, 2023; Langer et al., 2022)
				Direct measurement of binding affinity	Interference of auto-fluorescent or quenching ligands	
				High throughput	Limited to slow kinetic measurements	
				Independent of mass change		
				Low sample consumption		
				Can use challenging proteins (IDPs, membrane proteins, etc.)

SPR (Olaru et al. 2015; Acharya et al. 2024), for instance, enables real-time analysis, giving kinetic parameters and affinity constants (de Mol and Fischer 2008). SPR detects small changes in the refractive index near a sensor surface, measured through reflected light intensity (Bakhtiar 2013). In a typical SPR setup, light at a specific resonance wavelength excites surface plasmons on a thin layer of noble metal (Kooyman 2008; Wijaya et al. 2011), where one of the binding partners is immobilised. The binding of the interacting molecule causes an increase in the refractive index, resulting in a shift in the resonance frequency. As such, it necessitates surface attachment of the protein to the sensor. Although a variety of chip surfaces and coupling chemistries are available (Löfås and Mcwhirter 2006), immobilisation potentially impacts binding site accessibility and alters conformations, thereby affecting binding characteristics (Kortt et al. 1997). Advantageously, these tethered protein biosensors can be reused many times once suitable conditions for regeneration have been found (Holdgate et al. 2013). Unfortunately, immobilisation can often be particularly detrimental, e.g. for IDPs, as it disrupts the conformational equilibrium. In the case of more difficult targets, such as membrane proteins, which are especially sensitive to the acidic surface of the assay chips (Cooper 2002), immobilisation can be problematic. Furthermore, mass transport effects and non-specific interactions often complicate kinetic interpretations (Myszka et al. 1998; Schuck and Zhao 2010), generally giving a reliable limit of binding affinity detection at around 500 µM (Hubbard 2023), limited by solubility, although weaker affinities have been reported down to 5 mM (Giannetti 2011). Additionally, SPR experiments require significant experimental optimisation and proper treatment of surface effects during data analysis, which can be time-consuming. An example that particularly affects drug development is buffer additives (Giannetti 2011), such as detergents and dimethyl sulphoxide (DMSO; a common solvent for small molecules), which can cause artefacts in measurements. The SPR instrumental setup is complex, using microfluidics and pneumatics, and requires regular maintenance beyond user-level services and training (Giannetti 2011), increasing associated costs.

Biolayer interferometry (BLI) (Murali et al. 2022; Jug et al. 2024) is a related optical biosensor-based technique that analyses changes in the interference of white light reflected from a layer of immobilised protein on the biosensor tip and an internal reference layer, also providing kinetic and affinity data (Concepcion et al. 2009). Like SPR, BLI requires surface immobilisation of one interaction partner, which can introduce similar issues related to altered accessibility and conformational changes. The measurements are fast, increasing its throughput compared with SPR (Weeramange et al. 2020); however, BLI is also often not suitable for fragment screening, as the small target molecules may not have sufficient mass to allow for a distinguishable shift (Abdiche et al. 2008)—a problem that exists for SPR but is more pronounced for BLI. While BLI is generally simpler to operate than SPR, it still demands careful experimental design and optimisation, with surface-related artefacts that must be carefully managed during data analysis.

Isothermal calorimetry (ITC) is unparalleled in providing a complete thermodynamic profile through directly measuring heat changes during molecular binding (Ladbury & Chowdhry 1996). ITC is performed label-free and in solution, giving important information for studying time-dependent changes in ligand–protein engagement, important in late-stage drug development (Copeland et al. 2006). The calorimetric approach measures heat change upon binding by titrating one binding partner into a sample cell, which contains a constant amount of the other binding partner. However, to generate a sufficiently strong heat signal, it demands large amounts of pure sample (at micromolecular concentration ranges), which can be costly and is less suitable for high-throughput screening, especially as each titration takes 30–60 min (Lewis and Murphy 2005). For fragment screening or weak interactions, ITC is very impractical, with a theoretical limit in the low nanomolar range for direct *K*_*d*_ measurement (Leavitt and Freire 2001). Additionally, high levels of training are required for the use and maintenance of the equipment (Lewis and Murphy 2005).

NMR, which can be ligand- or protein-observed, can be used to qualitatively check if a ligand is bound by detecting changes in the characteristics of the reference spectra (Lamoree & Hubbard 2017; Y. Li & Kang 2017). It can detect binding affinities from covalent to as weak as 10 mM. Despite this, it is limited in throughput and requires significant amounts of protein (10 s of mg), which can be very costly and time-consuming.

Protein-observed NMR uses stable isotope-labelled protein, either ^15^N or ^13^C, usually in heteronuclear single quantum correlation spectroscopy (HSQC) (Lamoree & Hubbard 2017; Lepre 2011; Lepre et al. 2004). In drug discovery, human proteins are required to be expressed in eukaryotic cells (insect or mammalian) due to size and post-translational modifications. This significantly limits the capability of isotope labelling patterns at reasonable costs (Gossert and Jahnke 2016). Protein-observed NMR can provide affinity data using ligand titration and information on the ligand-binding site if the spectrum is assigned, and it easily distinguishes nonspecific binding effects. However, HSQC has significant limitations in protein size (upper limit of around 40 kDa) due to slower tumbling and broadening of NMR peaks observed above this size, which is a major limitation in therapeutic targets. Ligands must be analysed individually, and measurements can take several minutes per spectrum, so it is significantly limited in throughput.

Ligand-observed NMR usually uses three different 1-dimensional ^1^H NMR experiments—saturation transfer difference (STD), water-ligand-observed gradient spectroscopy (LOGSY), and Carr–Purcell–Meiboom–Gill sequence (CPMG) with a competition element (Lepre 2011; Lamoree and Hubbard 2017). Reference NMR spectra of each individual compound in the buffer of the target protein are required. Mixtures of fragments can be screened, usually 4–10, known as cocktailing. This requires more library preparation as it has the risk of interference in the spectra between compounds, making it harder to identify hits. Following from this, ligand-observed NMR requires limited assay development provided the target is suitably soluble, with no labelling or immobilisation requirements. Despite the possibility of automation in the process, where peaks can be automatically picked and compared to references (Klukowski et al. 2018), manual examination of spectra is still often required due to the significant risk of artefacts.

Analytical ultracentrifugation (AUC) observes the concentration distribution pattern of macromolecules at high centrifugal field (Harding & Rowe 2010). AUC provides information about stoichiometry and affinity for weak to very strong interactions between proteins and/or DNA. However, AUC is limited in throughput and requires specific equipment.

Thermal shift assays (TSA), such as differential scanning fluorimetry (DSF) (Semisotnov et al. 1991), monitor changes in the protein melting temperature upon ligand binding (Brandts & Lin 1990; Gao et al. 2020; Lo et al. 2004) using a conventional real-time polymerase chain reaction (RT-PCR) instrument (Bruce et al. 2019). As such, it is an inexpensive and easy-to-use method. Conventional TSA works by tracking fluorescence of an environment-sensitive (solvatochromic) dye, such as the commonly used SYPRO Orange (Steinberg et al. 1996), binding to exposed hydrophobic regions upon protein unfolding. The use of the dye that interacts directly with the protein may affect the protein unfolding behaviours by promoting transitions to the unfolded state (Kranz and Schalk-Hihi 2011). Despite being able to detect extremely tight binders to a sub-picomolar affinity, it is common that weak binding fragments do not stabilise the proteins to a detectable level, with a limit of around 1 µM (Hubbard 2023), giving frequent false negatives (Schulz et al. 2013). Nano-DSF (Alexander et al. 2014) works similarly to TSA but relies on the intrinsic fluorescence of the protein (Chattopadhyay and Varadarajan 2019). As the protein unfolds with increasing temperature, tryptophan and tyrosine residues previously buried become exposed to a polar solvent, and the emission maximum experiences a bathochromic shift from 330 to 350 nm (Ghisaidoobe and Chung 2014). A shift in melting temperature indicates an interaction, making it powerful for high-throughput molecule screening (Pantoliano et al. 2001). For many compounds, ligand binding directly correlates with ligand affinity, so binding affinities can be obtained; however, it is derived from both binding interactions and the thermodynamics of protein unfolding (Gao et al. 2020), so false positives and negatives are common. Most proteins contain multiple tyrosine and tryptophan residues, not all of which are involved in binding; as such, excitation gives a high fluorescence background, which decreases the amplitude of the signal (Burstein et al. 1973). These residues are often buried in the hydrophobic core and not affected much by ligand binding. Autofluorescence of ligands often lies in the same range as the Trp/Tyr fluorescence and thus interferes (Owicki 2000).

Microscale thermophoresis (MST) (Jerabek-Willemsen et al. 2014) detects changes in fluorescence caused by molecular movement under a temperature gradient. MST can be used with fluorescence labelling or intrinsic fluorescence (less common) (Seidel et al. 2013). Binding affects thermophoretic mobility due to changes in size, charge, and hydration (Duhr and Braun 2006)—as such, MST is not reliant on a change in mass or size (Seidel et al. 2013). While solution-based and sensitive (requiring minimal material), MST is limited in throughput due to its capillary-based nature in the proprietary (Baaske et al. 2018) NanoTemper Technologies Monolith technology series (Baaske et al. 2010). A further complication for MST could be the thermal instability of the binding partners.

Fluorescence polarisation (FP)—also known as fluorescence anisotropy—is widely used in high-throughput settings, assessing changes in rotational diffusion (Owicki 2000; Hall et al. 2016; Kumar et al. 2024). FP requires the fluorescent labelling of one of the binding partners, usually a small-molecule ligand or DNA (Townsend et al. 2014), which is then excited with polarised light. Fast rotation of the ligand causes the emitted light to be depolarised; the rotation slows when the molecular weight of the complex increases after binding, so the emitted light remains polarised to a greater extent. FP is limited by the compound interference from the large dye, usually fluorescein, which can alter the ligand’s binding behaviour. It is less suited where the larger of the two interaction partners must be labelled, as it relies on a change in molecular size upon binding. As many small molecules are not fluorescent, FP is often used as a competition (displacement) assay, whereby a fluorescently labelled tracer is outcompeted by a ligand that binds to the same binding site (Huang 2003). As such, it does not directly detect binding, giving only IC_50_ values rather than *K*_*d*_ values. FP is prone to artefacts and is limited to molecules that bind orthosterically and thereby displace the tracer.

Förster resonance energy transfer (FRET) and time-resolved FRET (TR-FRET) observe an energy transfer between two fluorescently labelled partners (Kumar et al. 2024). In TR-FRET, this occurs between a long-lived donor fluorophore and a short-lived acceptor fluorophore (Degorce 2009). The requirement for both partners to be fluorescent makes screening of compound libraries difficult and assay development complex. FRET is significantly limited by a maximum distance between fluorophores of 10–100 Å (Stryer and Haugland 1967). The efficiency varies inversely with distance, placing significant limitations on applications, especially when working with large proteins or protein complexes.

Despite the utility of these technologies, the limitations underscore the need for techniques that preserve native-like conditions, require minimal sample, and are amenable to high-throughput formats with minimal assay development—vital to modern drug development processes. In contrast to these methods, newer platforms—such as the Dianthus system—emphasise high-throughput capacity, miniaturisation, and freedom from immobilisation. The Dianthus leverages ratiometric fluorescence detection (Baaske and Langer 2023) and thermal perturbation to analyse binding directly in solution, without requiring complex assay development or surface coupling. This makes it especially well-suited for early-stage discovery programs, fragment screening, and systems with challenging biophysical properties, such as intrinsic disorder or conformational plasticity. As the molecular targets in drug development grow more complex—often involving dynamic, multicomponent systems—conventional biophysical technologies are being supplemented or replaced by innovative approaches that maintain native biological context while enabling scale, speed, and reproducibility. Dianthus represents a shift in this direction, providing a method that combines sensitivity, flexibility, and automation-readiness to align with modern drug discovery demands.

## Principles of spectral shift analysis

### Fluorescent labelling

**Fig. 2:**
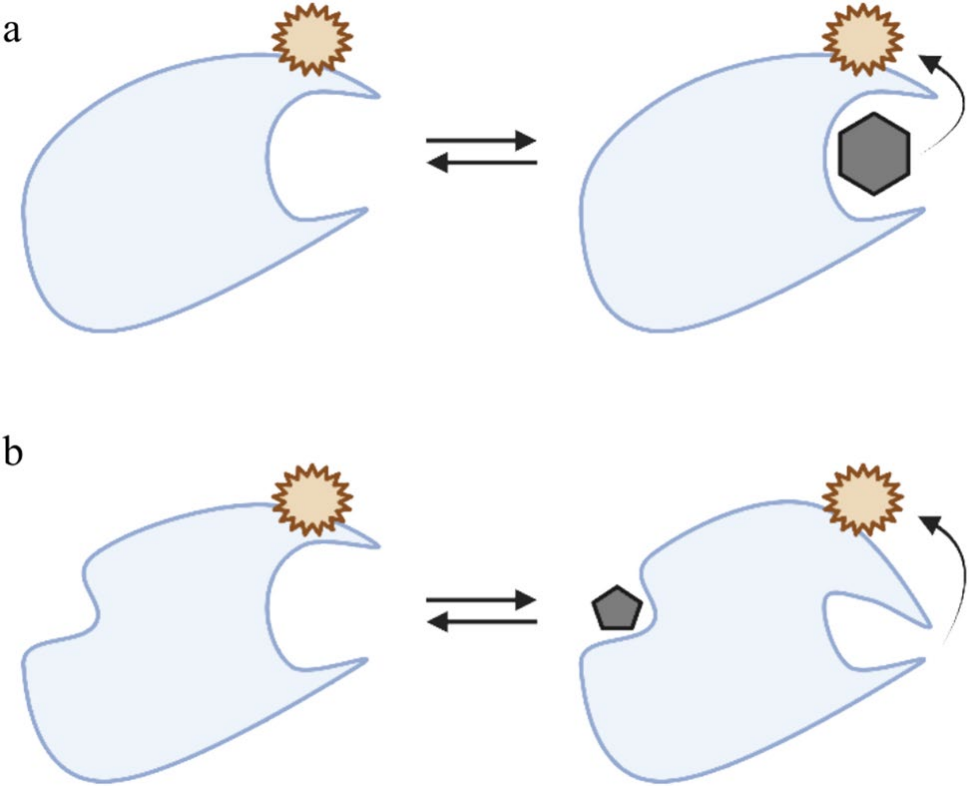
**a** Ligand binding affecting the fluorescent tag through proximity**. b** Ligand-induced conformational change (allostery) affecting the fluorescent tag (created in BioRender; https://BioRender.com/cmmdjrj)

Environment-sensitive fluorophores that reemit light upon excitation provide a powerful tool to report on changes in the local microenvironment of proteins. Upon ligand engagement, photophysical changes occur (dos Santos Rodrigues et al. 2023). Fluorophores in their ground state absorb incident light across a range of wavelengths centred around a characteristic excitation peak, promoting them to an excited electronic state. Upon relaxation to the ground state, they emit a photon of lower energy—a process modulated by vibrational relaxation and influenced by the surrounding microenvironment. The energy lost through non-radiative pathways shapes the emission spectrum and gives rise to the Stokes shift (Lakowicz 2006a). In cases where the intrinsic protein fluorescence is not sufficiently altered by ligand binding, dual-emission extrinsic dyes can be covalently attached to the protein via specific residues or tags. These dyes tend to have larger Stokes shifts and quantum efficiencies, meaning that they are much brighter than intrinsic fluorophores, reducing the required target concentrations and enabling detection of even picomolar affinities (Windsor and Raines 2015). Additionally, as previously mentioned, intrinsic fluorophores, such as aromatic amino acid residues, are often less responsive to binding-induced changes, making extrinsic dyes both more cost-effective and sensitive. Dyes emitting in the red spectral range (around 660 nm (Langer et al. 2022)), such as the RED-NHS dye from NanoTemper Technologies, are particularly advantageous in small-molecule screening. These longer wavelength emissions avoid interference from autofluorescence and background signals from dust (Schneider et al. 2013). Upon ligand binding, local changes near the fluorophore—such as alterations in polarity, surface hydrophobicity, and electrostatic interactions (Demchenko 2010), as well as temperature sensitivity—can affect its emission profile through broadening, shifts, and narrowings (Mayer‐Wrangowski and Rauh 2015; Sindrewicz et al. 2019). These changes may arise from direct ligand contact (Fig. 2a), induced conformational changes, as shown in Fig. 2b (e.g. amino acid side chain, loop, or domain movement), or alterations in surface charge and hydrophobicity. In some fluorophores, *cis–trans* isomerisation is the underlying mechanism: the fluorophore exists in distinct ground-state isomers with distinct spectroscopic properties (Chen et al. 2018). Ligand binding modulates local environment rigidity, affecting rotational freedom and thereby shifting the population between isomeric forms.

Supported labelling strategies (RED 2nd Generation), developed specifically for spectral shift and TRIC, have increased sensitivity, enabling the use of very low (down to single-digit nM) concentrations of the labelled target protein and allowing accurate determination of even sub-nM *K*_*d*_ values (Windsor and Raines 2015). These include covalent attachment via NHS-esters (targeting lysines), maleimide chemistries (targeting cysteines), non-covalent via tris-NTA labelling (for His-tagged proteins), non-covalent labelling for biotinylated targets, covalent benzylguanine labelling (for SNAP-tagged proteins), and non-covalent labelling of antibodies or antibody fragments containing the human Fc region. These protocols are fast (typically less than an hour), and some can be carried out even with unpurified samples, in cell lysate, or other complex bioliquids. NanoTemper provides online tools to facilitate the calculation of protein concentration and labelling efficiency (https://nanotempertech.com/user-tools/dol-calculator/). Far-red fluorescent fusion proteins can also be used, though they may exhibit lower quantum yields and greater photobleaching (light-induced degradation causing irreversible changes to their fluorescence) than chemical fluorophores.

### Theoretical background of spectral shift

**Fig. 3:**
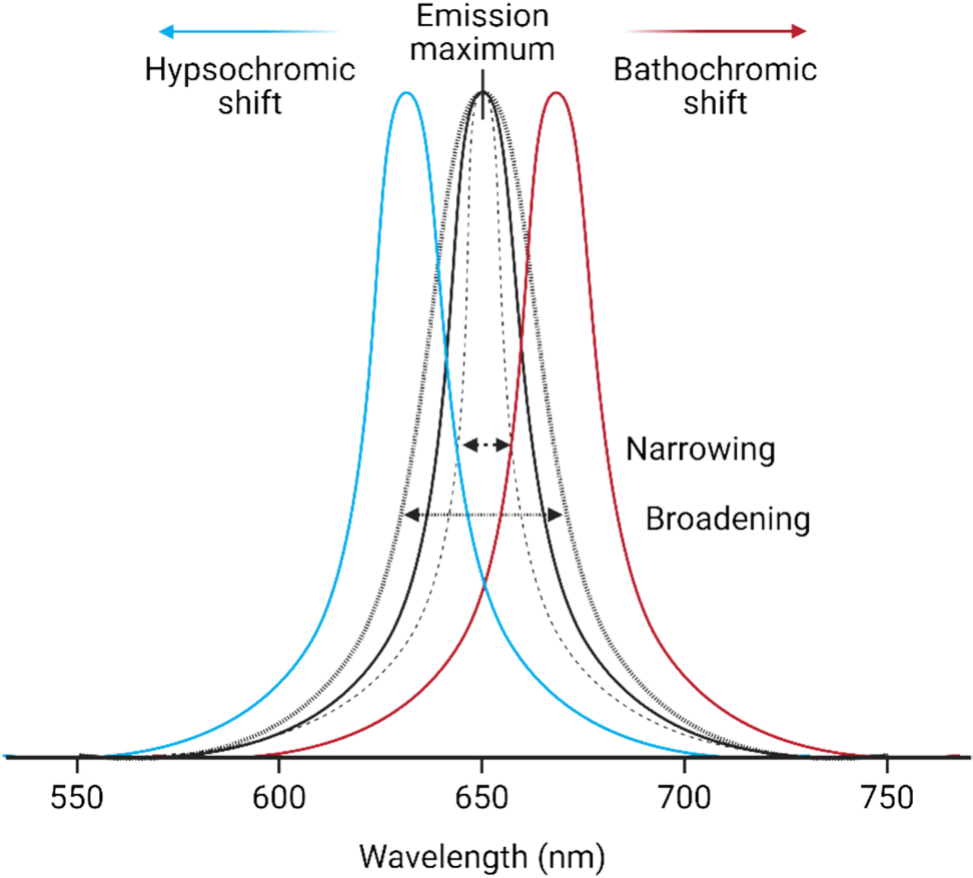
Emission profile changes through narrowings, broadenings, or spectral shifts (created in BioRender; https://BioRender.com/earf6xi)

Spectral shift assays, also known as wavelength (*λ*) ratiometry, exploit the environment sensitivity of a fluorophore to monitor ligand binding through changes in the emission spectrum (*F*(*λ*^em^)), as shown in Fig. 3, caused by different intra- and intermolecular relaxations. In the ratiometric technique, dual-wavelength detection at 650 and 670 nm (bracketing the fluorophore’s local emission maximum) and excitation at 590 nm is used.

The ratiometric technique avoids the limitations of intensity-only measurements which are prone to error, e.g. due to intrinsic ligand fluorescence, measurement noise (e.g. fluctuations in intensity of light source or variation of voltage at photodetector), non-specific effects such as binding of labelled molecules to surfaces, or masking of very small intensity changes behind pipetting errors (Demchenko 2014). Frequent false positives and negatives are introduced by measurements of absolute fluorescence intensities, and real binding events can be obscured (Resch-Genger et al. 2005). The ratiometric approach mitigates this, as it is not reliant on absolute fluorescence intensities (Langer et al. 2022). Previous implementations of ratiometric fluorescence measurements have been performed in other contexts using standard spectrofluorometers or plate readers (Pomorski et al. 2013; Demchenko 2014), which often lack the resolution for small shifts due to their broad spectral range. These setups, typically run in cuvettes or multi-well plates, require large amounts of material at higher concentrations, increasing cost and decreasing throughput. The observed spectral shift can be quantitatively described by the Lippert–Mataga equation (Eq. 1)(Lippert 1955), which relates the magnitude of the spectral shift (Stokes shift, Δ*λ*_SpS_) to differences in the dipole moments between excited (*µ*_*e*_) and ground states (*µ*_*g*_) of the fluorophore, the Onsager effective molecular cavity volume (*a*^3^), and the solvent polarity function (Δ*f*):

Equation 1 Lippert–Mataga equation describing spectral shift (Lippert 1955)

$$\Delta {\lambda }_{\text{SpS}}={\left[2 \bullet \frac{{\left({\mu }_{e}-{\mu }_{g}\right)}^{2}}{hc{a}^{3}} \bullet \Delta f+k\right]}^{-1}$$

**Fig. 4:**
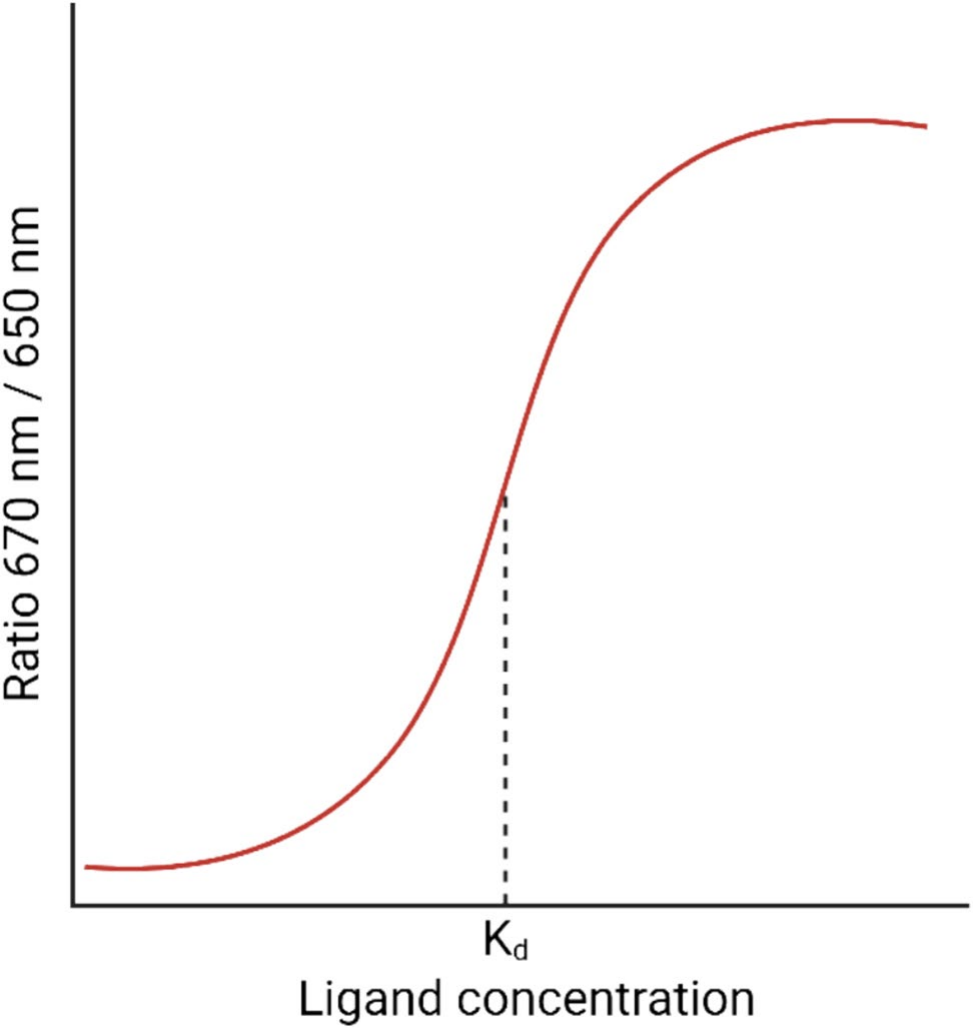
The plotting of the intensity ratios at two wavelengths against the ligand concentration determines *K*_*d*_ (Pomorski et al. 2013) (created in BioRender; https://BioRender.com/gcwxbrr)

From dose–response curves (Pomorski et al. 2013)-where fluorescence ratios are plotted against the ligand concentration (Fig. 4)-dissociation constants (*K*_*d*_) can be determined, typically assuming 1:1 stoichiometry binding according to the law of mass action (Langer et al. 2022).

The direction of the binding curve has no influence on the numerical value of the detected *K*_*d*_, but some qualitative information about conformational changes around the dye can be deduced. NanoTemper Technologies’ dyes exhibit negative solvatochromism, in which the ground state of the dye is more stabilised in polar environments than its excited state, opposite to most fluorophores. A hypsochromic (blue) shift typically indicates a more polar environment, while bathochromic (red) shifts, as shown in Fig. 3, reflect increased hydrophobicity and displacement of the hydration water shell around the dye. Fluorescence-based detection is central to Dianthus assays, typically requiring one of the binding partners to be labelled with a far-red fluorophore (matching the Dianthus filterset), such as Cy5-labelled nucleic acids or peptides.

### Orthogonal technique of temperature-related intensity change

**Fig. 5:**
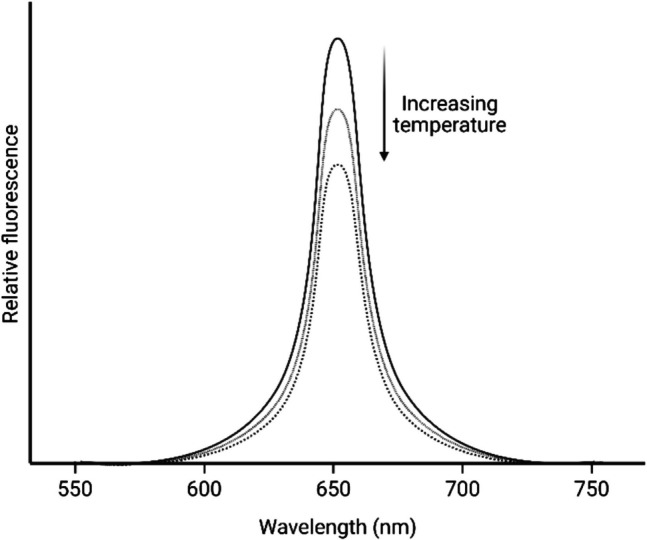
Temperature-related intensity changes of fluorescence (created in BioRender. https://BioRender.com/2v32ri7)

The TRIC technique offers a complementary method for detecting ligand binding by exploiting the thermal sensitivity of fluorophores (Baaske et al. 2010), as shown in Fig. 5. In this approach, a focussed infrared laser at 1480 nm induces a rapid localised temperature increase (up to 10 K), primarily through water absorption—achieved without physical contact (thus reducing contamination risk) or the need for thermally conductive plates. Fluorescence is monitored before and in the seconds following heating, capturing shifts in fluorophore behaviour between bound and unbound states. The rate of heating by the laser is generally faster than the rate of thermal destabilisation, making the method applicable even to thermally unstable targets.

**Fig. 6:**
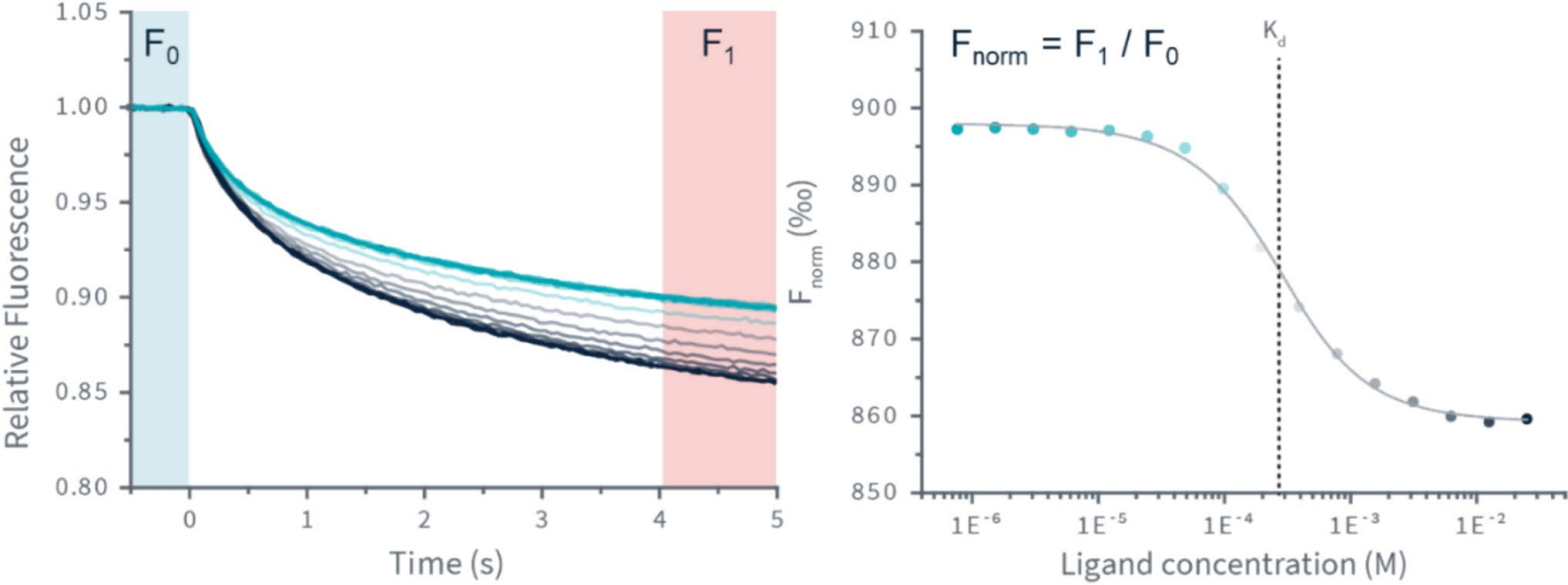
**a** TRIC trace of relative fluorescence against time from DI.Control: each line represents the change of relative fluorescence at a defined ligand concentration over time, with *F*_0_ (before heating) and *F*_1_ (set time after heating). **b** The plotting of *F*_norm_ values (ratio of fluorescence after heating to before heating) against ligand concentration allows *K*_*d*_ determination. This figure is provided by NanoTemper Technologies, with permission

TRIC focuses on the T-jump region, also used in MST, but is adapted here for higher throughput and lower sample consumption. Two key parameters are measured: *F*_0_, the initial fluorescence at ambient temperature (to assess sample homogeneity), and *F*_1_, the response amplitude at different ligand concentrations. The difference in normalised fluorescence (*F*_norm_) between bound and unbound states reflects the ligand binding affinity (Fig. 6). Users can choose to analyse data at different timepoints, *F*_1_ (e.g. 1, 3, or 5 s post-heating), as this can significantly affect the amplitude (López-Méndez et al. 2021), especially when the baseline signal is weak but increases at elevated temperatures.

A dose–response curve can be generated—plotting *F*_norm_ against ligand concentration—to determine the binding affinity. Importantly, TRIC enables real-time detection of problematic samples. Aggregated proteins produce fluctuating fluorescence signals as particles traverse the detection volume, which are automatically flagged by the Dianthus software. These fluctuations, common after repeated freeze–thaw cycles, can compromise assay reliability as molecules lose function. Autofluorescence and quenching by the ligands remain potential confounders in red-channel detection (Lakowicz 2006b). However, empirical studies by NanoTemper Technologies suggest that initial fluorescence changes below 20% (NanoTemper Technologies) typically do not interfere with TRIC signals and thus *K*_*d*_ fitting, and the software algorithm adjusts for deviations within this threshold (Langer et al. 2022). If the variation exceeds this threshold, the compound’s emission spectrum should be measured independently to assess autofluorescence and quenching effects. If necessary, the compound may be repurposed as the reporter molecule in place of a fluorescent target when a non-fluorescent variant of the target is unavailable.

### The Dianthus screening platform

**Fig. 7:**
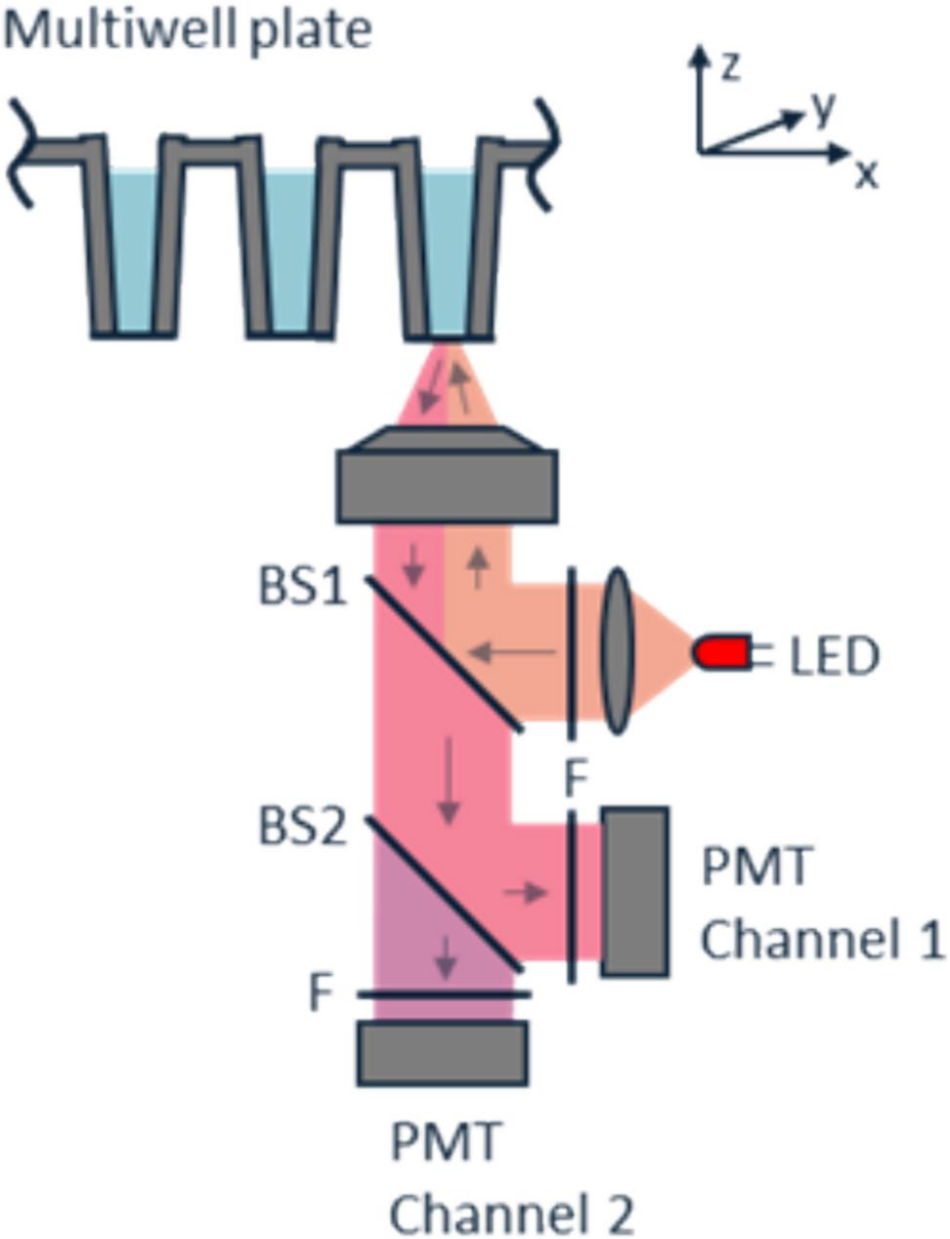
Experimental epifluorescence setup (Baaske & Langer 2023). This figure was provided by NanoTemper Technologies, with permission

The isothermal experimental setup for spectral shift experiments employs devices from the Dianthus series, as shown in Fig. 7 (Baaske & Langer 2023). The amber LED excites samples with monochromatic light at 592 nm, with power adjustable to achieve the desired fluorescence signal. This wavelength corresponds to the secondary absorption peak of near-infrared fluorophores. Unlike other light sources such as xenon lamps, LEDs do not emit infrared, so no heat filter is required to maintain isothermal conditions (Lakowicz 2006c). The excitation light is filtered through a bandpass optical filter made of low-autofluorescence glass centred at 593 nm before being reflected by a longpass 622 nm dichroic mirror (BS1). The light is then focused onto the sample using a lens (Baaske and Langer 2023). Two fixed wavelengths are detected simultaneously via dual-emission optics with an extended red multi-alkali photocathode containing head-on photomultiplier tubes (PMT) with borosilicate glass windows (Langer et al. 2022). PMTs provide enhanced sensitivity and reduced noise (Lakowicz 2006a). Emitted light passes through a longpass 660 nm dichroic mirror to split the beam (BS2) (Baaske & Langer 2023) and through bandpass emission filters (*F*) for < 660 and > 660 nm to clean up the fluorescence signal before reaching the respective detectors (Langer et al. 2022). Even minor emission peak shifts cause notable changes in the 670/650 ratio, with the system able to detect even sub-nanometer shifts. This setup (e.g. a linear focused dynode chain in PMT reduces transit time spread and improves time response) allows detection to occur within 50 ms or less, offering high temporal resolution, so that identical disturbances affect both detected wavelengths equally.

The Dianthus is a plate-based, microfluidics-free affinity screening platform developed by NanoTemper Technologies. It combines two complementary biophysical methods-spectral shift and TRIC-to measure molecular interactions under controlled equilibrium conditions without requiring immobilisation. The platform detects a broad range of binding affinities, from picomolar to millimolar, making it suitable for screening weakly binding fragment libraries as well as analysing more challenging interactions, such as multimeric complexes, covalent ligands, and intrinsically disordered proteins. It provides high-quality data with minimal assay development, making it suitable for stages of drug discovery from hit identification to lead optimisation. In particular, it can be integrated into automated workflows via Google Remote Procedure Call, facilitating high-throughput screening. The orthogonal nature of spectral shift and TRIC enhances confidence in detecting true binding events and increases versatility, especially in challenging cases such as low-affinity or transient interactions. The system supports both 384- and 1536-well formats, with full-plate spectral shift data acquisition completed in 35 and 7 min, respectively, making it highly suitable for high-throughput applications. Proprietary black polystyrene plates with a specialised well geometry and anti-absorptive coating prevent photobleaching, interference by autofluorescence, and non-specific binding. To ensure optimal readout, care must be taken to avoid introducing dust, scratches, or air bubbles, and adequate mixing (i.e. > 15 cycles) is critical during sample preparation (NanoTemper Technologies).

The platform operates at low nanomolar concentrations with minimal sample volumes (20 or 7 µl per well), preserving precious material and accommodating aggregation-prone targets, such as IDPs. Its fluorescence detection system is robust in realistic screening environments across common biological buffers and additives like DMSO-provided background fluorescence remains within acceptable limits (less than 50% of the signal from the labelled target (NanoTemper Technologies)). NanoTemper Technologies offers a dedicated Buffer Exploration Kit (Neumann et al. 2019) with integrated software support for optimisation. Low concentrations of detergent (below the critical micellar concentration) are often included to reduce non-specific adsorption and aggregation. As is typical for fluorescence-based assays, the excitation and emission characteristics can vary depending on buffer composition and pH, and buffer screening is recommended during assay development. Ideally, the buffer conditions reflect the physiological relevance of the protein target to improve translation of results to cellular and in vivo assays (Renaud et al. 2016). Both techniques are compatible with complex biological samples, including cell lysates, serum, and whole blood (Baaske et al. 2010; Wienken et al. 2010), allowing early assessment of target engagement in physiological conditions—important for preclinical validation.

Alongside MST, the MonolithX instrument (NanoTemper Technologies) also offers spectral shift technology. While both systems enable immobilisation-free binding affinity measurements, they cater to different experimental needs based on throughput and application focus. The Monolith is lower throughput—handling only 24 samples per run (Baaske and Duhr, 2014)—and capillary-based, requiring more liquid handling. As such, the Dianthus serves as a powerful tool for rapid high-throughput applications across multiple stages of drug discovery, from primary hit identification with single-dose screening through to lead optimisation with dose–response.

## The experimental setup

The basic Dianthus experiment is very fast. It includes the plate setup: the labelled protein (or labelled DNA/RNA) (called *target* in DI.Control software) at an approximate concentration (starting point 10 × lower than expected *K*_*d*_, if not known expect 1 µM as a starting point) is mixed with the binding partner (*ligand*) at an exact maximum concentration 50 × of the expected *K*_*d*_. The buffer for all solutions in the Dianthus experiment must be identical, including any additives such as detergents to prevent adsorption or DMSO to aid ligand solubility. The labelled protein is prone to surface attachment and aggregation; therefore, vortexing should be avoided. In the first part of the experiment, a *binding check* verifies that a spectral shift occurs. Three wells contain only protein, and three wells contain the mixture of protein and ligand at the highest concentration. The plate needs to be handled carefully, as the plastic film on the bottom is sensitive to scratches and fingerprints, which can compromise data collection. The plate is centrifuged to remove air bubbles (2 min at 1000 × *g*). Prior to the experiment, the wells are scanned in the X, Y, and Z directions to locate the centre of each well, and to detect evaporation, aggregate formation (which sediments and accumulates at the bottom of the well), air bubbles, and mixing inconsistencies. In the *binding check* measurements, a signal of > 0.005 and a signal-to-noise ratio > 5 should be observed. If the scans are very scattered, the labelling may be insufficient, or the protein concentration may be too low. Once a spectral shift is confirmed, the *binding affinity* experiment is performed. This involves measuring the spectral shift at two wavelengths for mixtures containing at least 16 different ligand concentrations with the target (e.g. protein) at a constant concentration. Plotting the ratio of emission intensities at the two wavelengths (670 nm/650 nm) against the ligand concentration using DI.Screening Analysis produces the titration curve of the binding pair, with the inflection point corresponding to the *K*_*d*_.

## Examples

### Protein–ligand interactions: sensitivity for weak binders.

**Fig. 8:**
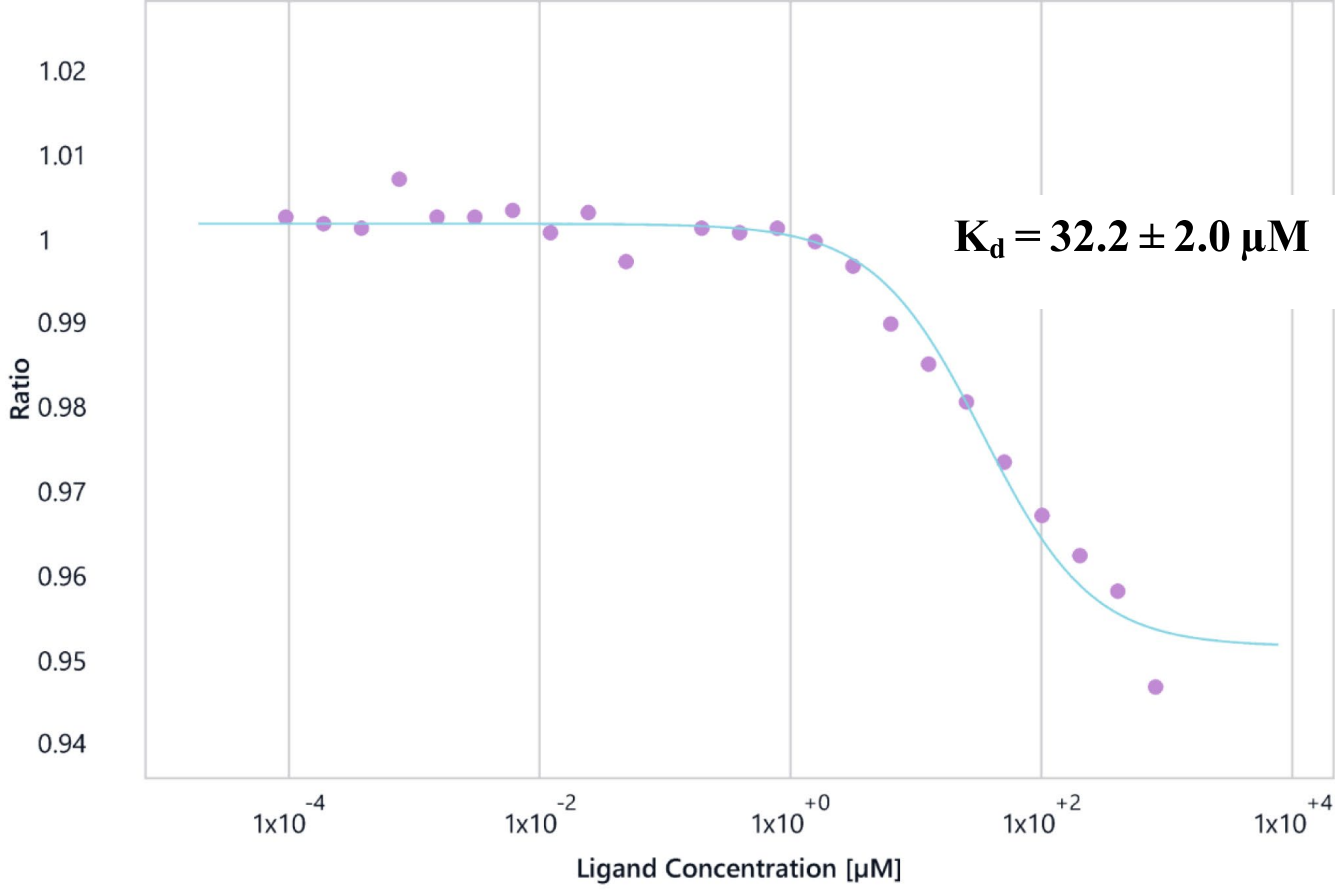
HEWL-NAG spectral shift binding curve produced by DI.Screening Analysis with NAG at a maximum concentration of 800 µM and lysine-labelled HEWL at 50 nM. Signal-to-noise ratio: 18.6; saturation of the binding curve: 95.9%

Small-molecule ligands binding to protein targets represent the most common type of interaction studied in early drug discovery. Numerous studies have utilised spectral shift technology for this purpose (Langer et al. 2022), although only a few report the use of the Dianthus platform. Zian et al. (2025) describe high-throughput screening (HTS) for antiviral inhibitors targeting the 3-chymotrypsin-like protease of SARS-CoV-2, the virus responsible for the recent global pandemic. Classic model systems, such as the binding of oligosaccharides to hen egg white lysozyme (HEWL), serve as benchmarks for characterising ligand recognition and affinity. We therefore evaluated HEWL-NAG (*N*,*N*′,*N*″-triacetylchitotriose) interaction, which is well characterised. The binding constant was previously determined by ITC experiments to be 11.1 ± 1.1 µM and by laser electrospray mass spectroscopy to be 6.8 ± 1.5 µM (Archer et al. 2018). Our own SPR experiments yielded a *K*_*d*_ of 10.9 ± 1.1 µM. The Dianthus experiment was performed four times using solutions from the same NAG half-dilution series (maximum concentration 800 µM) with 50 nM HEWL, resulting in a *K*_*d*_ of 32.2 ± 2.0 µM (see Fig. 8).

**Fig. 9:**
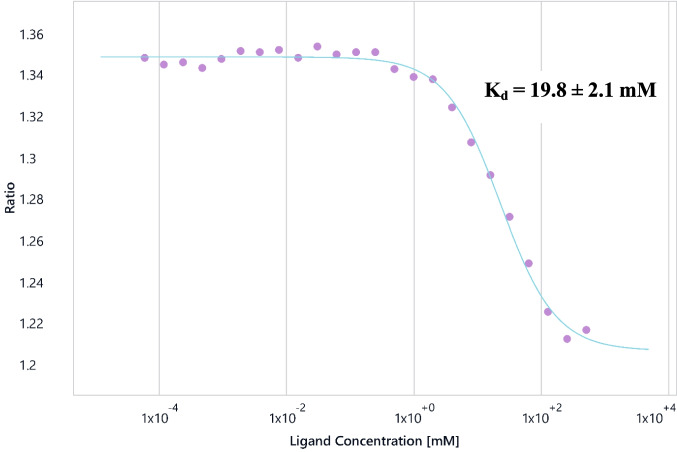
*Tc*CS-l-cysteine binding curve with l-cysteine at a maximum concentration of 500 mM and lysine labelled *Tc*CS at 10 nM. Signal-to-noise ratio: 39.1, saturation of the binding curve: 95.6%

The ability of the Dianthus to detect even weak binders makes it suitable for fragment screening as well as PROTAC development. As an example for a low-affinity interaction, we characterised the interactions between *Trypanosoma cruzi* cysteine synthase (*Tc*CS) and l-cysteine (Gasparikova 2025). Unsurprisingly, l-cysteine binds only weakly to *Tc*CS, as it is produced by the enzyme from *O*-acetyl l-serine (OAS) (Sowerby et al. 2023). The Dianthus experiment was performed four times using solutions from the same l-cysteine dilution series (maximum concentration: 500 mM) with 10 nM *Tc*CS, resulting in a *K*_*d*_ 19.8 ± 2.1 mM (see Fig. 9).

Membrane protein analysis is a significant challenge in drug discovery due to their instability outside native lipid environments. Recent innovations utilising polymer-encapsulated nanodiscs (e.g. PoLiPa and DIBMA-based systems) provide a native-like lipid environment that preserves structure and function. In a case study with the adenosine A2A receptor, PoLiPa nanodiscs enabled robust fragment screening using spectral shift, confirmed by nDSF, and results were consistent with known antagonist affinities (https://www.domainex.co.uk/drug-discovery-case-studies/adenosine-a2a-receptor-novel-biophysical-fragment-screening). Likewise, detailed protocols utilising spectral shift in the MonolithX have been applied to study the interaction between bacterial SecA ATPase and the SecYEG nanodisc complex (https://support.nanotempertech.com/hc/en-us/article_attachments/32159955499025; https://support.nanotempertech.com/hc/en-us/article_attachments/32149995601425), confirming high-affinity binding (*K*_*d*_ ~ 18–37 nM). In parallel, spectral shift experiments using the Dianthus have also been successfully applied to detergent-solubilised membrane proteins, as demonstrated in the investigation of ligand binding to the serotonin transporter (SERT) in micelles, revealing distinct ion-dependent affinities(Kalenderoglou et al. 2025) (https://pmc.ncbi.nlm.nih.gov/articles/PMC11838746/#F13). Together, these examples highlight how nanodisc- and micelle-stabilised membrane proteins, when combined with spectral shift, enable biophysical interrogation of protein–protein and protein–ligand interactions with minimal perturbation of native structure.

### Competition assays

The ability to perform competition binding assays provides valuable insights into binding site occupancy and mechanism of action, and can be utilised in situations where the protein of interest is not amenable to labelling or immobilisation. While FP has been a common approach, it typically requires high protein concentrations and may be sensitive to assay conditions. Spectral shift assays can provide an alternative, as demonstrated in studies using fluorescent ATP-competitive probes to evaluate kinase binding in near-native states (https://www.domainex.co.uk/drug-discovery-case-studies/spectral-shift-competition-assay-robust-and-versatile-assay-assess). By titrating inhibitors against a constant concentration of kinase and fluorescent probe, high-resolution binding assays were generated, yielding IC_50_ values across a wide dynamic range from low nanomolar to high micromolar. These values correlated well with orthogonal biochemical assay results, confirming the sensitivity and translatability of spectral shift results across assay formats. One study using the Dianthus spectral shift binding assay (Chandler et al. 2025) demonstrates the disruption of the protein–protein interaction (PPI) between BRISC and SHMT2 by the novel BLUE compound FK-171-C.

### Protein–protein interactions: Binary and multimetric complexes

**Fig. 10:**
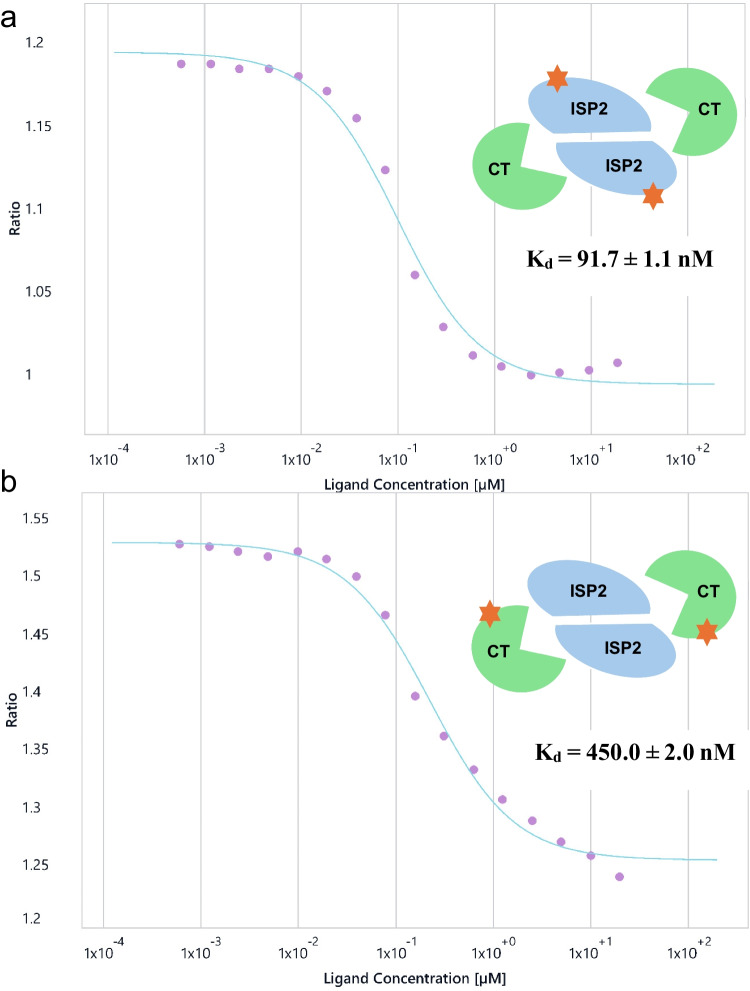
**a** Binding of bovine α-chymotrypsin to 10 nM labelled *Lm*ISP2, using a half-dilution series with a maximum concentration of 19 µM. **b** Binding of *Lm*ISP2 to 10 nM labelled bovine α-chymotrypsin, using a half-dilution series with a maximum concentration of 20 µl

PPIs present added complexity due to their often transient and low-affinity nature (Lu et al. 2020; Scott et al. 2016). This is particularly important given the increase in the focus on biologics (https://web.cas.org/marketing/pdf/CASBIOENGWHP101215-CAS-IP-Biologics-Innovation-White-Paper.pdf) and PPI disruptions (Lu et al. 2020). Small-molecule drug discovery targeting PPIs to inhibit complex formation has proved very challenging due to large, shallow binding sites with distant interaction regions (Renaud et al. 2016). An illustrative example is the interaction between *Leishmania major* protein inhibitor of serine protease 2 (*Lm*ISP2) and bovine chymotrypsin (CT), studied recently by us. Here, the nuanced interface formed between host and pathogen proteins is reflected. Sensitive detection of such binary PPIs in solution is essential for understanding binding kinetics and thermodynamics without perturbation. In a first set of experiments, a constant 10 nM concentration of RED-NHS-labelled *Lm*ISP2 was used to bind unlabelled bovine CT, with a maximum concentration of 19 µM, as shown in Fig. 10a. Spectral shift analysis yielded a *K*_*d*_ of 91.7 ± 1.1 nM. Similarly, a constant 10 nM concentration of RED-NHS-labelled CT was used to bind unlabelled *Lm*ISP2, with a maximum concentration of 20 µM, as shown in Fig. 10b, yielding a spectral shift of 450.0 ± 2.0 nM. Saturation was 100% for all data, and the signal-to-noise ratio was above 20.

*K*_*d*_ differences may be due to labelling artefacts in the case of labelled CT, as Lys93 of chymotrypsin is close to the binding interface and Lys175 is involved in a hydrogen bond, both of which may be disturbed by the labelling of the amines with RED-NHS. The literature reports that *Lm*ISP2 inhibits human chymotrypsin with a *K*_*i*_ of 19 ± 4.2 nM (Eschenlauer et al. 2009). *K*_*d*_ provides a more direct measure of affinity than *K*_*i*_, which only accurately reflects the binding constant when the kinetic mechanism is correctly identified, particularly if the mechanism is not ideal competitive inhibition, which was assumed in this study for *K*_*i*_ calculation (H. Cheng 2004; H. C. Cheng 2001).

Even more challenging are multimeric protein complexes, such as the ternary assemblies formed by PROTACs or molecular glues, which simultaneously bind a target protein and an E3 ubiquitin ligase, representing a growing area of research (D. Li et al. 2022). Cooperativity, usually quantified by the α factor (Schnatwinkel et al. 2025), is a key determinant of degradation efficacy (Bondeson et al. 2018; de Castro & Ciulli 2021). Functional activity is dependent on the simultaneous engagement of both partners as a ternary complex, with interactions formed between the E3 ligase and the target protein via de novo protein contacts (Gadd et al. 2017). Historically, PROTACs have been evaluated using proximity-dependent assays, such as TR-FRET or ALPHA (Mostofian et al. 2023), but these methods lack the sensitivity to resolve weak, transient interactions characteristic of the binary complex states (Mostofian et al. 2023; Shaik et al. 2024; Zorba et al. 2018). Spectral shift provides a highly sensitive technique. Application notes (https://support.nanotempertech.com/hc/en-us/article_attachments/27088417843089; https://support.nanotempertech.com/hc/en-us/article_attachments/27088389021457) from NanoTemper Technologies demonstrate the use of spectral shift in the Monolith X to monitor PROTAC-mediated ternary complex formation via fluorescently labelled VCB or BRD2^BD2^ in titrations with MZ1. These studies reported a binary *K*_*d*_ of 61.2 nM and a ternary *K*_*d*_ of 1.83 nM, yielding a cooperativity of 33.4-comparable to values obtained from SPR and ITC (Gadd et al. 2017; Roy et al. 2019). The expected hook effect was observed at low BRD2^BD2^ concentrations, demonstrating the known dynamics of ternary complex formation. Complementary data on BRD3^BD2^ and other PROTACs reaffirm the platform’s utility in ternary complex quantification (https://support.nanotempertech.com/hc/en-us/article_attachments/27088428001809; https://support.nanotempertech.com/hc/en-us/article_attachments/27088427999633; https://www.domainex.co.uk/sites/default/files/2025-01/Domainex%20Poster_The%20Application%20of%20Spectral%20Shift%20to%20Drug%20Discovery%20Projects.pdf). In another example, the Dianthus platform has been used to estimate cooperativity in ternary complexes involving molecular glues for FKBP12 and MAPRE1 (Schnatwinkel et al. 2025). In a recent study, Schnatwinkel et al. (2025) demonstrate that spectral shift analysis enables direct retrieval of intrinsic cooperativity (*α*) values from single titration experiments without the need for multiple binary references. By titrating a series of macrocyclic molecular glues against fluorescently labelled FKBP12 in the presence or absence of MAPRE1, compounds with favourable ternary stabilisation profiles and *α* values exceeding 10-indicative of strong cooperative binding-could be identified. The inclusion of an aggregate checker in the Dianthus software helps mitigate the risk of artefactual signals caused by protein aggregation, which is especially important when handling multicomponent systems. As antibody–antigen complexes demonstrate very high affinity, the Dianthus PICO must be used to generate precise *K*_*d*_ values. Typically, the antigen is labelled at a 1–5-nM concentration. The following application papers describe antibody characterisation with spectral shift methods: https://support.nanotempertech.com/hc/en-us/article_attachments/32158609581457andhttps://support.nanotempertech.com/hc/en-us/article_attachments/31797390990993.

### Protein–nucleic acid interactions

In general, protein–DNA or protein–RNA interactions can be studied by labelling the protein, as described above, or by using Cy5-labelled nucleic acids. In this application (https://www.domainex.co.uk/sites/default/files/2025-01/Domainex%20Poster_The%20Application%20of%20Spectral%20Shift%20to%20Drug%20Discovery%20Projects.pdf), a labelled-RNA fragment screen was used for spectral shift assays. Targeting RNA in drug discovery can provide an alternative therapeutic strategy for undruggable proteins by preventing their synthesis. Here, 112 hits were studied further, and three fragments with affinities < 600 µM were identified. A recent application demonstrated the use of spectral shift to characterise binding between the bacterial transcriptional repressor Mlc and a DNA hairpin, serving as a model for its operator sequence (https://support.nanotempertech.com/hc/en-us/article_attachments/31797205447185). Cy5-labelled BoxP1 Hairpin DNA was titrated with increasing concentrations of Mlc dimer, yielding a *K*_*d*_ of 26.4 ± 1.2 nM, in agreement with previously obtained data of 24 nM from SPR and 29.8 nM from ITC (Witte 2015). These findings underscore the utility of spectral shift technologies for analysing protein–nucleic acid complexes.

## Applications in drug discovery

Here, we outline key applications across the drug discovery pipeline, from specific high-throughput screening to lead optimisation. The combination of sensitivity, speed, and quantitation accuracy makes the Dianthus platform a valuable technology in pharmaceutical development.

### High-throughput screening

The capabilities of the Dianthus extend to conventional high-throughput screening of large chemical libraries-a critical step in early-phase drug discovery. Drug development campaigns often employ a process in which either many drug-like molecules (HTS) or fragments (FBDD) are rapidly assayed against specific biological targets to identify hits. The primary goal of HTS is to use formats that increase throughput (to at least a few thousand compounds per day) and reduce assay volume, thereby lowering costs, while maintaining sensitivity and reliability. HTS libraries typically consist of many compounds (often millions) with molecular weights of 300–450 Da (R. Hubbard 2023; Reymond et al. 2010). A large focus has been placed on HTS due to the rise of combinatorial chemistry, which has exponentially increased the size and diversity of screening libraries (Appell et al. 2001). The Dianthus platform is compatible with 384- or 1536-well plate formats (the latter available only for the uHTS Dianthus), enabling high-throughput screening capabilities. The recently introduced uHTS Dianthus can process a full 1536-well plate using spectral shift in just 8 min. Its immobilisation-free approach streamlines assay development while retaining physiological relevance, and its rapid, high-throughput nature makes it ideal for large-scale screening campaigns.

### Fragment-based drug discovery

Fragment-based screening is increasingly being favoured over HTS (Erlanson et al. 2016), as it uses significantly smaller library sizes while covering a more diverse chemical space (Lamoree & Hubbard 2018). These libraries typically have reduced functional group complexity but enhanced solubility (Acharya et al. 2024). They often contain 500–2000 molecules (R. E. Hubbard & Murray 2011) with molecular weights of 120–250 Da (Lamoree & Hubbard 2018) and are designed to target multiple binding pockets beyond the active site. Despite weaker individual affinities, fragment hits exhibit higher ligand efficiencies (LE)-affinity per atom (Hopkins et al. 2004)-making them excellent starting points for optimisation. These low-affinity binders can be elaborated or combined into potential leads with higher overall affinity. Detecting such interactions requires high-sensitivity platforms that operate independently of molecular weight and are not affected by the high fragment concentrations typically required. Consequently, many high-throughput biochemical platforms used in HTS fail in this context (Renaud et al. 2016). The Dianthus platform’s sensitivity reduces false negatives, as the smaller compounds employed in fragment screens often exhibit weaker affinities. It can detect binding affinities even in the millimolar range, independent of the molecular mass of the interaction partners (SPR is limited to 90 Da; Acharya et al. 2024). The experiments are primarily limited by the solubility of the fragments tested; fortunately, fragments generally exhibit higher solubility than larger compounds. The Dianthus addresses the challenges of FBDD by measuring interactions in solution without the need for immobilisation, thereby preserving the native behaviour of both ligands and protein targets.

**Fig. 11:**
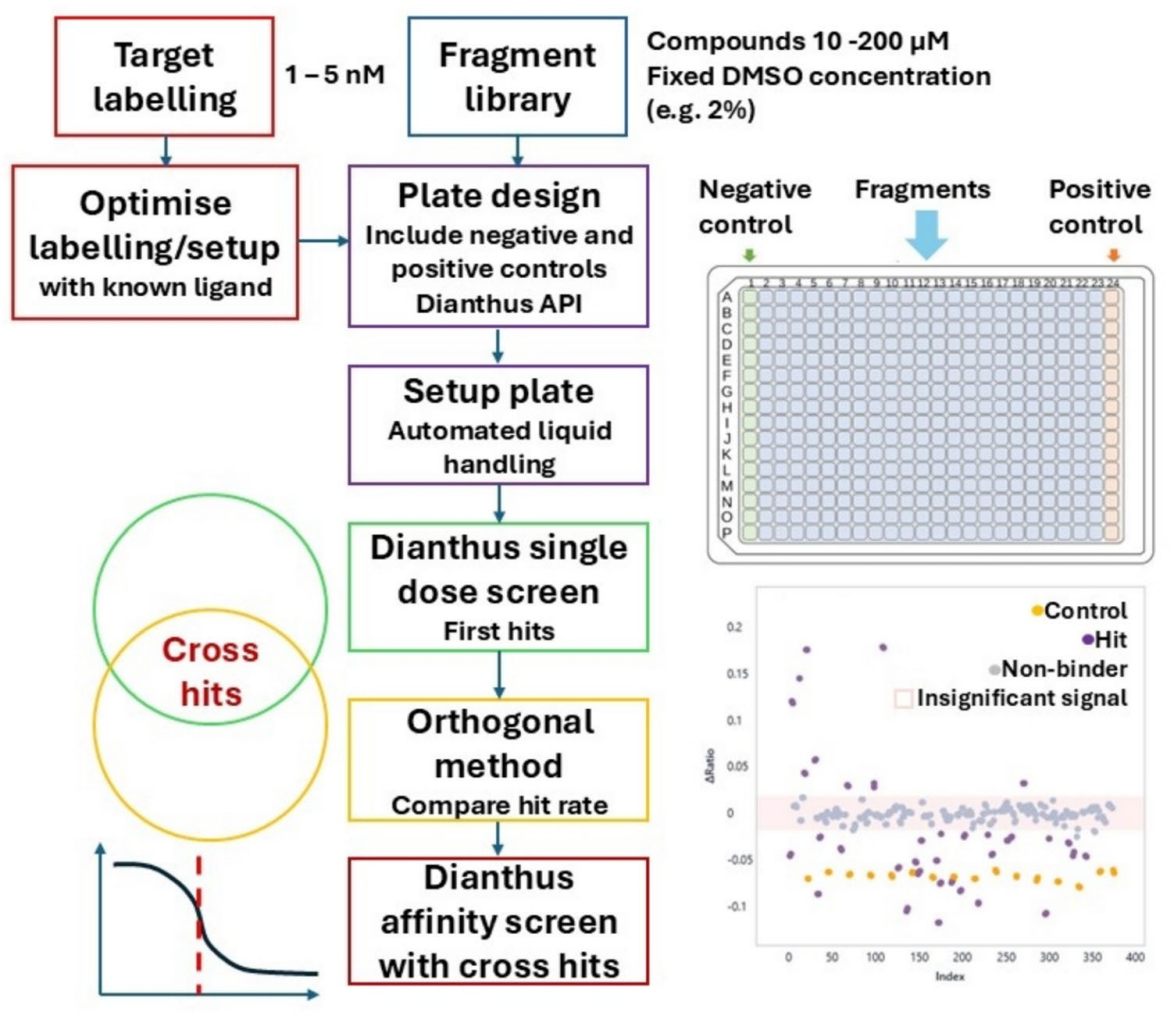
Schematic workflow of fragment screening with Dianthus. The DI.Screening Analysis output is the intellectual property of NanoTemper Technologies and is for informational use only; it may not be shared or reproduced without prior written consent

The fragment screening experiment using the Dianthus platform is illustrated in Fig. 11. The initial steps involve protein labelling and fragment library preparation, as previously described. Buffer conditions should be identical for both protein and ligand solutions; a fixed concentration of detergents or DMSO is recommended, although this depends on ligand/fragment solubility and protein stability. Labelling and experimental setup should be optimised, ideally using a known ligand, which can be achieved through *binding check* and *binding affinity* experiments. Finally, the plate setup should be designed to include space for negative controls (labelled protein in buffer) and positive controls (mixture of labelled protein and known ligand at high concentration). Fragments can be tested either once or multiple times in single-dose experiments. When targeted libraries are used, the success rate for this type of screening can be > 10%. The number of hits is generally project-dependent and can be < 1% when maximum diversity libraries are employed. The DI.Screening Analysis software evaluates the single-dose spectral shift experiments, and the Z’-factor-a statistical quality parameter that incorporates control data (Zhang et al. 1999)-should be applied. The Δratio of the intensity shifts at two wavelengths is plotted against the fragment sequence number to distinguish hits from non-binders. Cross hits are confirmed using an orthogonal method such as X-ray crystallography, SPR, NMR, or DSF, and are then forwarded to *binding affinity* experiments for further evaluation. HTS and fragment screening using the TRIC method have been described (Schulte et al. 2021; Jeridi et al. 2021). In a recent study, Alexander et al. (2014) investigated inhibitors of the cancer drug target KRAS-GDP/GppNHp using SPR and SpS/TRIC.

### Hit-to-lead optimisation: Structure–activity relationship and affinity ranking

Following the identification and characterisation of initial hits, the drug discovery process progresses to the hit-to-lead phase, where compounds (typically fewer than 100; Renaud et al. 2016) are refined and prioritised based on potency, selectivity, and drug-like properties. Multi-parameter hit-to-lead optimisation requires increasingly detailed data on compound binding behaviour. At this stage, precise measurement of binding affinity is essential, as even small improvements in molecular interactions can significantly influence downstream efficacy and pharmacokinetics.

Chemodynamic analysis (Renaud et al. 2016) can be performed to evaluate different buffer conditions (such as pH, ionic strength, and additives) and their influence on the interaction. This provides valuable insights into the forces influencing these interactions during lead optimisation and assesses the robustness of the interaction for future relevance. The orthogonal TRIC technology facilitates validation, streamlining the process, as the binding of various hits often requires validation by one or more orthogonal biophysical methods (R. Hubbard 2023). Validating targets is essential to remove false hits before investing significant time and resources. Solution-based assays, such as those employed by the Dianthus, are particularly well-suited for this phase of development. Their high sensitivity allows accurate quantification of binding affinities across a broad dynamic range, capturing subtle differences between closely related analogues. This capability is critical for supporting structure–activity relationship (SAR) studies, where iterative chemical modifications are evaluated for their impact on target engagement. Such incremental improvements often correlate directly with downstream pharmacological outcomes, making them essential for rational lead development. This was recently exemplified by Wittenburg et al. 2025), where application of the spectral shift assay using the Dianthus detected minor modifications to the USP7-targeting warhead, resulting in subtle shifts in binding affinity—differences that would likely be overlooked using lower-resolution techniques.

## Conclusions

Biophysical methods for determining binding affinities offer the advantage of not relying on biological activity and providing a more direct means to study biomolecular interactions. The use of solution-based binding assays across the drug discovery continuum-from initial mechanistic studies to screening and lead refinement-demonstrates their broad applicability. By combining sensitivity, throughput, and experimental simplicity, these technologies have become indispensable tools for addressing the growing complexity of therapeutic discovery. NanoTemper’s Dianthus platform, utilising spectral shift technology, represents a significant advancement in the study of macromolecular interactions. Enhanced signal-to-noise ratios, increased sensitivity, reduced susceptibility to insoluble aggregates, and its high-throughput format make spectral shift methods well-suited for modern drug discovery pipelines. In summary, the key advantages of this approach include non-immobilisation, high throughput, independence from mass changes, low sample consumption, and versatility in studying diverse binding partners. Spectral shift offers a high-throughput/low-sample consumption approach for primary screening, as well as a higher-content, lower-throughput method for characterising drug candidates, making it a valuable tool in drug discovery. Targets that were previously challenging in small-molecule drug discovery-including protein–protein interactions, multi-protein complexes, and IDPs-can be effectively studied using spectral shift assays. As therapeutic discovery advances toward increasingly complex modalities, platforms like the Dianthus will be pivotal for understanding macromolecular interactions with both precision and speed.

### SI1. Materials and methods

Experiments were performed in PBS, supplemented with 0.005% Tween-20, using a Dianthus NT23 (Nano) in 384-well plates (16 × 24). All proteins have been labelled employing their lysine residues with the RED-NHS 2nd Generation kit (NanoTemper Technologies) according to the supplied protocol. For binding partners twofold dilution series were prepared. 20 µL of a 1:1 mixture of labelled protein and binding partner dilution were added into each well for 16 or 24 dilutions.

### SI1.1. HEWL-NAG

A twofold dilution series of NAG (N, N’, N’’-Triacetylchitotriose, Sigma T2144, maximum concentration 800 µM) was used for a binding affinity experiment in the Dianthus DI.Control with constant 50 nM labelled HEWL (Melford L38100) concentration.

### SI1.2 *Tc*CS-L-cysteine

*Trypanosoma cruzi* cysteine synthase, *Tc*CS was recombinantly produced as described in Sowerby et al 2023 and labelled. L-cysteine (Merck 30089) was twofold diluted in a series starting with 500 mM maximum concentration for a binding affinity experiment with *Tc*CS at 10 nM.

### SI1.3 *Lm*ISP2-CT

*Leishmania major* ISP2 was recombinantly expressed from the plasmid pGL1179 (ISP2/pET15b) in *E.coli* as described in Eschenlauer et al 2009*.* The protein was purified with IMAC methods and subsequently polished with size exclusion chromatography using a Cytiva HiLoad 16/600 Superdex 200 pg column in Hepes buffer. CT (Bovine α-chymotrypsin, Sigma C3142) was used as binding partner for labelled *Lm*ISP2 (10 nM) with the maximum concentration of 19 µM in a dilution series for a binding affinity experiment. Subsequently CT was labelled and the concentration kept at 5 nM for a binding affinity experiment with *Lm*ISP2 used as a binding partner (dilution series with maximum concentration 130 µM).

## Data Availability

No datasets were generated or analysed during the current study.

## Abbreviations

BLI: Biolayer interferometry
BRD2^BD2^: Bromodomain of bromodomain-containing protein 2
BS: Beam splitter
CPMG: Carr–Purcell–Meiboom–Gill sequence
CT: Chymotrypsin
DDB1: DNA damage binding protein 1
DMSO: Dimethyl sulphoxide
DSF: Differential scanning fluorimetry
F: Filter
FBDD: Fragment-based drug discovery
FP: Fluorescence polarisation
FRET: Förster resonance energy transfer
gRPC: Google remote procedure call
HEWL: Hen egg white lysozyme
HSQC: Heteronuclear single quantum correlation spectroscopy
HTS: High-throughput screening
IC_50_: Half maximal inhibitory concentration
IDP: Intrinsically disordered protein
ISP2: Inhibitor of serine protease 2
ITC: Isothermal calorimetry
K_d_: Equilibrium dissociation constant
LED: Light-emitting diode
LOGSY: Water-ligand-observed gradient spectroscopy
MST: Microscale thermophoresis
NAG: *N*,*N*′,*N*″-Triacetylchitotriose
nDSF: Nano-differential scanning fluorimetry
NMR: Nuclear magnetic resonance
PBS: Phosphate-buffered saline
PMT: Photomultiplier tubes
PPI: Protein–protein interaction
PROTAC: Proteolysis targeting chimera
SAR: Structure–activity relationship
SPR: Surface plasmon resonance
SpS: Spectral shift
STAT3: Signal transducer and activator of transcription 3
STD: Saturation transfer difference
TcCS: *Trypanosoma cruzii* cysteine synthase
TPD: Targeted protein degradation
TRIC: Temperature-related intensity change
TSA: Thermal shift assay
VCB: Von Hippel–Lindau elongin C–elongin B
